# Male-killing mechanisms vary between *Spiroplasma* species

**DOI:** 10.3389/fmicb.2022.1075199

**Published:** 2022-11-28

**Authors:** Hiroshi Arai, Maki N. Inoue, Daisuke Kageyama

**Affiliations:** ^1^United Graduate School of Agricultural Science, Tokyo University of Agriculture and Technology, Fuchu, Japan; ^2^Institute of Agrobiological Sciences, National Agriculture and Food Research Organization (NARO), Tsukuba, Japan

**Keywords:** *Spiroplasma*, male-killing, symbiosis, evolution, endosymbionts, *Homona magnanima*, *spaid*

## Abstract

Male-killing, a male-specific death of arthropod hosts during development, is induced by *Spiroplasma* (Mollicutes) endosymbionts of the Citri–Poulsonii and the Ixodetis groups, which are phylogenetically distant groups. *Spiroplasma poulsonii* induces male-killing in *Drosophila melanogaster* (Diptera) using the Spaid toxin that harbors ankyrin repeats, whereas little is known about the origin and mechanisms of male-killing induced by *Spiroplasma ixodetis.* Here, we analyzed the genome and the biological characteristics of a male-killing *S. ixodetis* strain *s*Hm in the moth *Homona magnanima* (Tortricidae, Lepidoptera). Strain *s*Hm harbored a 2.1 Mb chromosome and two potential plasmids encoding Type IV effectors, putatively involved in virulence and host–symbiont interactions. Moreover, *s*Hm did not harbor the *spaid* gene but harbored 10 ankyrin genes that were homologous to those in other *S. ixodetis* strains. In contrast to the predominant existence of *S. poulsonii* in hemolymph, our quantitative PCR assays revealed a systemic distribution of strain *s*Hm in *H. magnanima*, with particularly high titers in Malpighian tubules but low titers in hemolymph. Furthermore, transinfection assays confirmed that strain *s*Hm can infect cultured cells derived from distantly related insects, namely *Aedes albopictus* (Diptera) and *Bombyx mori* (Lepidoptera). These results suggest different origins and characteristics of *S. ixodetis*- and *S. poulsonii*-induced male-killing.

## Introduction

In arthropods, maternally inherited endosymbiotic microbes frequently interact with the hosts in a mutualistic or a parasitic manner. Male-killing (MK), male-specific death in insects during development, is one of the reproductive manipulations induced by various intracellular bacteria, microsporidia, and viruses (Duron et al., 2008; Werren et al., 2008; Kageyama et al., 2012; Fujita et al., 2021). MK leads to the advantage of female siblings and is considered a selfish strategy of the intracellular microbes that promotes their spread and survival in nature (Hurst, 1991; Hurst and Jiggins, 2000; Hornett et al., 2006). The genus *Spiroplasma* (class: Mollicutes) are the most studied bacteria that induce MK in diverse insects (Anbutsu and Fukatsu, 2011; Lo et al., 2015; Harumoto and Lemaitre, 2018; Binetruy et al., 2019). *Spiroplasma* are small, helical, and motile bacteria that include commensal, pathogenic, and mutualistic species and have a diverse host range, including plants and animals (Regassa and Gasparich, 2006; Duperron et al., 2013; Viver et al., 2017; He et al., 2018). Phylogenetically, the MK *Spiroplasma* strains are clustered into the Citri–Poulsonii group (harbored by *Drosophila* flies and lacewings) (Williamson and Poulson, 1979; Hayashi et al., 2016) and the Ixodetis clade (harbored by ladybugs, butterflies, moths, and aphids) (Hurst et al., 1999; Simon et al., 2011; Tabata et al., 2011; Smith et al., 2016).

The molecular mechanisms underlying *Spiroplasma*-induced MK have been mostly investigated using *S. poulsonii*–*Drosophila* systems (Harumoto and Lemaitre, 2018). *S. poulsonii* strain MSRO induces MK in *Drosophila melanogaster* by a toxic protein androcidin (Spaid) harboring ankyrin repeats that damage the male X chromosome (Harumoto and Lemaitre, 2018). In contrast, information regarding the mechanism underlying MK induced by the members of the Ixodetis group is limited. The *spaid* gene is conserved among *S. poulsonii* strains (Harumoto and Lemaitre, 2018; Gerth et al., 2021), whereas whether the *S. ixodetis* group uses Spaid as an MK factor is unknown. The genus *Spiroplasma* exhibits high genomic flexibility and dynamic evolution of various toxin loci, such as Spaid and ribosome-inactivating protein (RIP) (Hamilton et al., 2016; Ballinger et al., 2019; Gerth et al., 2021; Massey and Newton, 2022; Pollmann et al., 2022). Gnomic analyses have revealed dynamic *Spiroplasma* evolution driven by bacteriophage lysogenization (Ye et al., 1996; Carle et al., 2010; Ku et al., 2013) and by horizontal gene transfer (Mouches et al., 1984; Joshi et al., 2005). Virulence-associated genes are frequently exchanged between microbes sharing the same niche (Kent and Bordenstein, 2010; Wiedenbeck and Cohan, 2011). Likewise, *Spiroplasma* may have acquired MK genes by horizontal gene transfer because they often coexist with other endosymbionts, such as *Wolbachia* and *Rickettsia*, in the same host (Hurst et al., 1999; Majerus et al., 2000; Watanabe et al., 2012; Hayashi et al., 2016; Takamatsu et al., 2021). However, the horizontal gene transfer to *Spiroplasma* may be constrained by the unusual codon usage by *Spiroplasma* compared with other bacteria (notably, the use of UGA as a tryptophan rather than a stop codon; Lo et al., 2015). Although genomic studies on MK *S. poulsonii* have been done, comparative genomic analyses of other MK *Spiroplasma* species, such as *S. ixodetis*, are essential to infer the origin and evolution of the MK machinery.

In this study, we sequenced the genome of *S. ixodetis* strain *s*Hm that causes MK in the tea tortrix moth *Homona magnanima* (Tortricidae, Lepidoptera). Against the full-genome sequence of strain *s*Hm, we searched for genes encoding Spaid and RIP toxin homologs, as well as putative MK genes of other MK endosymbionts such as *Wolbachia* (Arai et al., 2020, 2022a) and Partiti-like virus Osugoroshivirus (OGVs) (Fujita et al., 2021) in *H. magnanima*. We also examined the propagation characteristics and infectivity of strain *s*Hm using quantification and transinfection assays. Finally, we argue that MK mechanisms and ecological characteristics are substantially different between *Spiroplasma* species.

## Materials and methods

### Rearing and sexing of *Homona magnanima*

To construct *S. ixodetis* sHm genome, we used the laboratory-maintained *Spiroplasma*-positive MK-inducing line (S+ line) of *H. magnanima* (Tsugeno et al., 2017). In the present study, we accidentally obtained a *Spiroplasma*-positive 1:1 sex ratio line (S+M+ line) as a subline of the S+ line. For every generation, the male moths picked up from the 1:1 sex ratio line, which had been confirmed negative for *Spiroplasma*, *Wolbachia*, and OGV (NSR line) (Takamatsu et al., 2021), were crossed with the female moths of the S+ and S+M+ lines as described by Arai et al. (2022a). The obtained larvae were reared using artificial diet SilkMate 2S (Nosan Co., Yokohama, Japan) at 25°C under a long photoperiod (16L:8D), i.e., till pupation. To eliminate *Spiroplasma* from the S+ line, the first instar larvae were reared with SilkMate 2S supplemented with 0.05% tetracycline (w/w) as described by Arai et al. (2019). Adult moths were sexed based on their morphology, and the hatched larvae and the unhatched pharate larvae (mature embryo) were sexed based on the presence or absence of the female-specific sex chromatin body (a condensed W chromosome), which was detected *via* lactic-acetic orcein staining (Arai et al., 2022a).

### *Spiroplasma* detection and quantification in *Homona magnanima*

Total DNA was extracted from the abdomen of female adults (0-day post eclosion), the whole body of larvae and pupae (0-day post molting), and dissected tissues of *H. magnanima* larvae (0-day post molting) using cell lysis buffer, as described by Arai et al. (2019). To detect *Spiroplasma*, a pair of *Spiroplasma*-specific primers was used to amplify RNA polymerase β subunit gene (*RpoB*), which is a single copy conserved gene in *Spiroplasma* spp., from the extracted DNA (adjusted to 50–100 ng/reaction) with EmeraldAmp MAX PCR Master Mix (TaKaRa Bio, Shiga, Japan); the primer sets are listed in Table 1. The PCR conditions were as follows: 35 cycles of 94°C for 30 s, 55°C for 30 s, and 72°C for 30 s, followed by 72°C for 7 min. β-Actin gene of *H. magnanima* was used as the control. To quantify *Spiroplasma* density, qPCR was performed using the extracted DNA, which was diluted to a concentration of 10 ng/μL with MilliQ water, *Spiroplasma RpoB* primers (Table 1), and KOD SYBR^®^ qPCR Mix (Toyobo, Osaka, Japan) in a LightCycler^®^ 96 system (Roche, Basel, Switzerland). The PCR consisted of 45 cycles of 98°C for 10 s, 60°C for 10 s, and 68°C for 30 s. Relative abundance of the gene was calculated using the expression of elongation factor 1a gene (*ef1a*) of *H. magnanima* as the control. *Spiroplasma* density (*RpoB* copies) and relative abundance (*RpoB*/*ef1a*) were calculated as described in Arai et al. (2019, 2022c).

**TABLE 1:** Sequences and related information of the primers used in this study.

Target	Gene	Primers sequences (5′–3′)	Product size (bp)	Annealing temperature (°C)	References
*H. magnanima*	β-Actin	297f:AACTGGGATGACATGGAGAAGATCTGGC	838	55	Tsugeno et al., 2017
		1139r: GAGATCCACATCTGCTGGAAGGTGGACAG			
	*HmEf-1a*	Hmef1a_F_val1_85: TTTCCAGGGTGGTTGAGCA	108	60	Arai et al., 2022c
		Hmef1a_R_val1_193: CCGTTAAGGAGCTGCGTCG			
	*COI*	LepF: ATTCAACCAATCATAAAGATATTGG	650	55	Hajibabaei et al., 2006
		LepR: TAAACTTCTGGATGTCCAAAAAATCA			
*Spiroplasma*	*RpoB*	HmSpiro_RpoB388qF: GCATACTCAACACCCGTACCA	95	60	This study
		HmSpiro_RpoB483qR: TGCTAACCGTGCTTTAATGGG			
		HmSpiro_RpoB155F: CGCCATCTTTCATCGAAGGTC	423	60	
		HmSpiro_RpoB578R ATTGTTGGACCAAACGAAGTTG			

### Genome sequence of the *Spiroplasma* sHm strain

For genome sequencing of strain *s*Hm, high molecular weight DNA was extracted from the egg masses of S+ line moths using Nanobind Tissue Big DNA Kit (Circulomics Inc., MD, USA) and used for library construction using Ultra-Long DNA Sequencing Kit (Oxford Nanopore Technologies, Oxford, UK) following the manufacture’s protocol. The constructed libraries were sequenced using ONT MinION flow cell (R 9.4.1) (Oxford Nanopore Technologies). The obtained reads were mapped to the *H. magnanima* reference genome (Jouraku et al., in preparation) with minimap2 (Li, 2018), and the non-mapped reads containing *Spiroplasma* reads were extracted with SAMtools v.1.9 (Li et al., 2009) and assembled using Canu 1.6 (Koren et al., 2017). The draft *Spiroplasma* genome (a circular main chromosome and plasmids) was annotated *via* BLASTn (NCBI nr database). The extracted DNA was also subjected to Illumina paired-end 150 bp sequencing (PE-150) at Novogene (Beijing, China). The Illumina data were used to polish the draft genome using minimap2 (Li, 2018) and Pilon v. 1.23 (Walker et al., 2014). Since no sequence changes were observed after the second polishing, the polished genome was considered as the complete genome of strain *s*Hm. The circularity of the *s*Hm genome was confirmed by BLASTn search, followed by manual deletion of overlapping sequence.

### Resequencing of the sHm strain in the S+ and S+M+ moth lines

S+ (MK line) and S+M+ *H. magnanima* lines (non-MK line) were used for DNA extraction as described by Arai et al. (2022a). The DNA extracted from *Spiroplasma* cells was amplified using whole genome amplification (WGA) by REPLI-g Mini Kit (Qiagen, Hilden, Germany), following the manufacture’s protocol. The WGA products, purified using AMPure XP beads (Beckman Coulter, Inc., CA, USA) and dissolved into TE buffer, were sequenced on Illumina platform (PE-150). The Illumina data assembled with unicycler (Wick et al., 2017) and Illumina raw read data were mapped to the *s*Hm reference genome using minimap2 (Li, 2018) to detect the genomic changes in the genome of *s*Hm in the S + M + line.

### Genome annotations and homology surveys

The constructed *s*Hm genome was annotated *via* DFAST (Tanizawa et al., 2018). Effector genes were further annotated using EffectiveDB (Eichinger et al., 2016). Functional analysis of proteins (i.e., domain predictions and Gene ontology annotations) was conducted using InterPro.^1^ Phage WO infections were annotated using PHASTER (Arndt et al., 2016). Protein homology between different *Spiroplasma* strains was analyzed using *S. apis* B31 (CP006682.1), *S. citri* strain BLH-MB (CP047437.1–CP047446.1), *S. syrphidicola* strain EA-1 (NC_021284.1), *D. melanogaster* endosymbiont *S. poulsonii* MSRO (CM020866.1–CM020867.1) (*s*Mel, MK strain, Masson et al., 2018), *Danaus chrysippus* (Nymphalidae) endosymbiont *S. ixodetis* (NZ_CADDIL010000001.1–NZ_CADDIL010000012.1) (*s*Da, MK strain, Martin et al., 2020), *Lariophagus distinguendus* (Pteromalidae) endosymbiont *S. ixodetis* (NZ_JALMUW010000001.1–NZ_JALMUW010000198.1) [*s*Dis, cytoplasmic incompatibility (CI) strain, Pollmann et al., 2022], and *Dactylopius coccus* (Dactylopiidae) endosymbiont *S. ixodetis* (JACSER010000001.1–JACSER010000358.1) (*s*Coc, non-MK strain, Vera-Ponce León et al., 2021) with OrthoVenn2.^2^ Homology of *s*Hm genes and proteins with *spaid* from strain *s*Mel (Harumoto and Lemaitre, 2018), ankyrin genes from *S. ixodetis* (Yeoman et al., 2019; Martin et al., 2020; Vera-Ponce León et al., 2021), and the *Wolbachia* MK candidate factor responsible for WO-mediated killing (Wmk, presumed helix-turn-helix transcriptional regulator, Perlmutter et al., 2019; Arai et al., 2022b) was evaluated using both BLASTn and BLASTp. Moreover, to verify whether MK microbes of *H. magnanima* carried conserved genes, the genes on the MK-associated prophage region WO*w*Hm-t76 of MK *Wolbachia w*Hm-t (Arai et al., 2022b) and those of the Partiti-like virus OGVs (Fujita et al., 2021) were compared to the *s*Hm genes using both BLASTn and BLASTp. Unique genomic features of the *s*Hm strain in the MK S+ and non-MK S+M+ *H. magnanima* lines were analyzed using GView^3^ and BV-BRC variation analysis service.^4^ Metabolic pathways of *S. poulsonii s*Mel and *S. ixodetis s*Hm were compared by using BV-BRC comparative analysis service (see text footnote 4). Phylogenetic trees of 16S rRNA gene and ankyrin genes of *Spiroplasma* strains were constructed by maximum likelihood with bootstrap re-sampling of 1,000 replicates using MEGA7 (Kumar et al., 2016). *Mycoplasma genitalium* G-37 (NR074611.1) was used as an outgroup.

### Transinfection assays

A fifth instar female larva was sterilized in 50% bleach (ca. 3% sodium hypochlorite) for 10 min, in 70% ethanol for 10 min, and dissected in IPL-41 Insect Medium (Gibco, Carlsbad, CA, USA) supplemented with 10% (v/v) fetal bovine serum (FBS). Malpighian tubules of the dissected larva were transferred to flasks containing either the *Bombyx mori* NIAS-Bm-aff3 (aff3) cell line (Takahashi et al., 2006) or the *Aedes albopictus* NIAS-AeAl-2 (AeAl2) cell line (Mitsuhashi, 1981) maintained in IPL-41 Insect Medium (Gibco) with 10% (v/v) of FBS. The cells and Malpighian tubules were co-cultured at 23°C. Fresh medium was supplied to the flask every 10 d. Purified cells centrifuged at 1,000 *g* for 2 min were used to analyze infections and titers of the transinfected strain *s*Hm in the cells. DNA extraction, PCR, and qPCR assays were performed as mentioned in section “Spiroplasma detection and quantification in *Homona magnanima*.”

### Statistical analysis

Sex ratio bias was assessed using Fisher’s exact test. *Spiroplasma* densities, male ratio in hatched larvae, and male ratio in unhatched pharate larvae were analyzed using either the Wilcoxon test or the Steel--Dwass test. All analyses were performed using R software v4.0^5^.

## Results and discussion

### *Spiroplasma ixodetis* strain sHm induced embryonic male death in *Homona magnanima*

**FIGURE 1:**
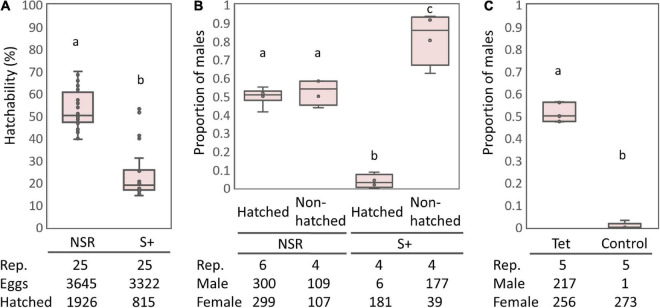
Egg hatching rates and sex ratios of the S+ line of *Homona magnanima.* **(A)** Egg hatching rates of 25 egg masses for each of the NSR and S+ lines (total 3,645 and 3,322 eggs, respectively). **(B)** Proportion of males among the hatched larvae and the unhatched pharate larvae. **(C)** Proportion of males of the subsequent generation after tetracycline treatment. Tet, tetracycline treatment; Control, non-treated control. The horizontal bar within the box represents the median. The upper and lower hinges of the box indicate upper quartile and lower quartile, respectively. Sample sizes are indicated below the panels. Different letters indicate significant differences between groups (Steel–Dwass test, *P* < 0.05).

The S+ line moths harboring *s*Hm exhibited lower egg-hatching rates than the NSR line (Figure 1A), which is consistent with the results from previous studies that *Spiroplasma* infection halved the egg hatching rates of *H. magnanima* (Tsugeno et al., 2017; Takamatsu et al., 2021). Cytogenetic sexing based on the presence or absence of a sex chromatin body (W chromosome) revealed that the sex ratio of hatched larvae was strongly biased toward females in the S+ line moths but not in the NSR line moths (*P* < 0.01, Figure 1B). In contrast, the sex ratio of unhatched pharate larvae (late-stage embryos) were male-biased in the *s*Hm-infected line (S+) (Fisher’s exact test, *P* < 0.01), confirming that *s*Hm killed male *H. magnanima* during embryogenesis. Moreover, the elimination of *Spiroplasma* by tetracycline treatment resulted in non-biased sex ratios in the subsequent generation (*P* < 0.01, Figure 1C).

### Genome sequence and genetic characteristics of male-killing *Spiroplasma ixodetis* strain sHm

**FIGURE 2:**
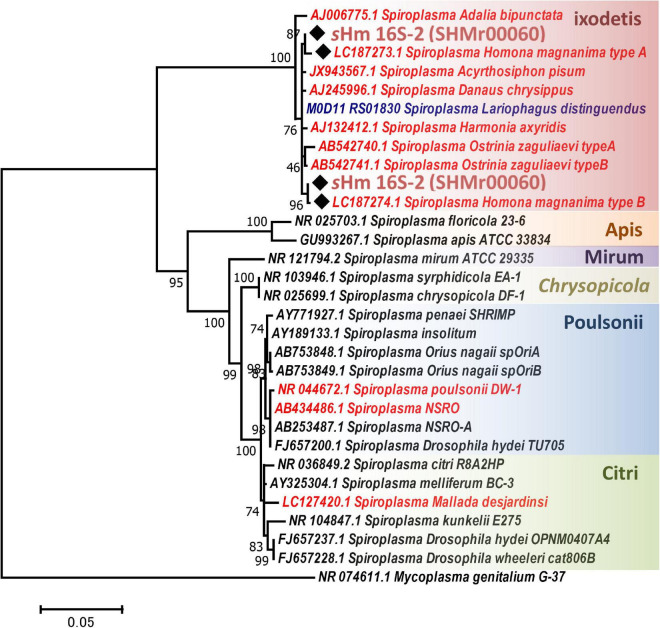
Phylogenetic tree of *Spiroplasma* strains based on 16S rRNA gene sequences. Phylogenetic tree based on 16S rRNA gene sequences of strain *s*Hm and other *Spiroplasma* strains (retrieved from NCBI database) using maximum likelihood method based on the Tamura–Nei model with 1,000 bootstrap replicates. Accession numbers are shown along with the operational taxonomic units (OTUs). Samples highlighted with red and blue color fonts are MK and CI strains, respectively. Black diamonds indicate 16S rRNA sequences of the *s*Hm. The classification of *Spiroplasma* is based on the study by Paredes et al. (2015). *Mycoplasma genitalium* was used as the outgroup.

Both Illumina (816.3 Mb, 5,442,459 reads, and 150 bp average length) and Nanopore data (93.5 Mb, 25,820 reads, and 3,624 bp average length) were used to reconstruct a complete genome consisting of a circular main chromosome (2, 102,039 bp in length) and two circular potential plasmids [20,119 bp (pSHM_1) and 16,408 bp (pSHM_2)]. Previously, Tsugeno et al. (2017) reported two 16S rRNA gene variants cloned from *Spiroplasma*-infected *H. magnanima*, but they did not elucidate whether the two sequences were interoperonic polymorphs of a single isolate or they were derived from two different strains. The present study confirmed that *H. magnanima* was infected with the MK *S. ixodetis* strain *s*Hm that harbored two distinct 16S rRNA gene sequences in its genome (Figure 2). Moreover, *Spiroplasma* strains often encode multiple ribosomal RNA gene sets in their genome (Chang et al., 2014; Tsai et al., 2018; Vera-Ponce León et al., 2021).

Strain *s*Hm harbored a higher number of coding sequences (CDS; 2,886 CDS) than strains *s*Mel (1.9 Mb in genome size; 2,405 CDS; Masson et al., 2018) and *s*Da (1.7 Mb in genome size; 1,813 CDS; Martin et al., 2020; Table 2). Although plasmids often contain key accessory genes such as *spaid* of *s*Mel (Harumoto and Lemaitre, 2018), genes on pSHM_1 (*n* = 24) and pSHM_2 (*n* = 20) mostly encoded hypothetical or uncharacterized proteins (Supplementary Table 1). In addition, strain *s*Hm harbored 12 prophage regions (size: 6.6–14.8 kb) in its genome, which is consistent with previous reports that several phage sequences are found in *Spiroplasma* genomes (Bai and Hogenhout, 2002; Lo et al., 2013; Ramirez et al., 2021). Bacteriophages frequently carry virulence-associated genes that encode toxins (Waldor and Mekalanos, 1996; Brüssow et al., 2004). Recently, the mechanistic bases of *Wolbachia*-induced cytoplasmic incompatibility (CI) and MK have been attributed to phages (Beckmann et al., 2017; LePage et al., 2017; Perlmutter et al., 2019; Arai et al., 2022b). Besides, phages have also been implicated in the defense phenotype exhibited by bacteria against parasitoids, such as *Hamiltonella defensa* (Brandt et al., 2017). Therefore, it is possible that the phages of *s*Hm contribute to the manifestation of MK phenotype or confer fitness advantage on hosts by protecting the hosts from natural enemies.

**TABLE 2:** Genomic features of strain *s*Hm and other *Spiroplasma* strains found in insects.

Genome ID	*Spiroplasma ixodetis s*Hm	*Spiroplasma ixodetis s*Da	*Spiroplasma ixodetis* DCM	*Spiroplasma ixodetis s*Dis	*Spiroplasma poulsonii* MSRO
Main chromosome/contigs	1 (closed/circular)	12	353	198	1 (closed/circular)
Plasmids	2 (closed/circular)	NA	NA	3 (closed/circular)	1 (closed/circular)
Estimated genome size (Mb)	2.14	1.75	1.32	1.16	1.96
N50	2,102,039	265,779	7,774	14,219	1,938,611
G + C content (%)	25.1	23.7	24.16	24.3	26.3
CDS genes	2,886	1,813	1,371	1,175	2,405
rRNA (16S, 5S, 23S)	6 (2,2,2)	4 (1,2,1)	3 (1,1,1)	3 (1,1,1)	3 (1,1,1)
tRNA	27	27	27	27	31
Phenotype	MK^1^	MK^2^	non-MK^3^	CI^4^	MK^5^
Insect associated	*Homona magnanima* ^1^	*Danaus chrysippus* ^2^	*Dactylopius coccus* ^3^	*Lariophagus distinguendus* ^4^	*Drosophila melanogaster* ^5^

^1^Based on Tsugeno et al. (2017).

^2^Based on Martin et al. (2020).

^3^Based on Vera-Ponce León et al. (2021).

^4^Based on Pollmann et al. (2022).

^5^Based on Masson et al. (2018).

### sHm harbored putative virulence-associated factors but did not harbor sMel spaid toxin

Recently, Yeoman et al. (2019) and Vera-Ponce León et al. (2021) reported that *D. coccus*-infecting *S. ixodetis* (sCoc) harbored a *spaid* homolog and *Cephus cinctus* (Cephidae)-infecting *S. ixodetis* harbored seven *spaid* homologs. Our BLAST searches confirmed that *s*Hm did not harbor the *spaid* gene (Table 3), however, some of the ankyrin genes of *s*Hm were homologous to the alleged gene sequences of *s*Coc and Cephidae-infecting *S. ixodetis* (Table 4). It is likely that *S. ixodetis* do not harbor the *spaid* gene. The superficial homology could be due to the presence of conserved ankyrin repeats (Table 3). Similarly, the amino acid sequences of ankyrin proteins of *s*Hm (such as SHM_18920) showed partial homology to the ankyrin domain of Spaid from strain *s*Mel (N-terminal 200 amino acids) as per BLASTp search, but the complete amino acid sequences of the proteins of these two strains were not homologous (Table 3). Moreover, we also confirmed the absence of Spaid homologs in a MK *S. ixodetis* strain *s*Da by using BLASTp search. Gerth et al. (2021) reported that the Spaid homologs are conserved among *S. poulsonii* strains regardless of the MK phenotype. Because the spaid gene is not likely to be possessed by *S. ixodetis*, MK mechanisms may differ between *S. poulsonii* and *S. ixodetis* (i.e., having different causative genes).

**TABLE 3:** Homology between Spaid [1,065 aa] of strain *s*Mel and proteins of *Spiroplasma ixodetis* strains based on BLASTp search.

*Spiroplasma ixodetis* proteins	Identity	Aligned length	*s*Mel spaid		*Spiroplasma ixodetis*		*e*-value	Bit score	References
			Start	End	Start	End			
sHm (SHM_18920)	36.0	205	217	409	60	261	2.99E-29	111	This study
sDa (SPD_05340)	40.9	220	54	266	28	237	5.56E-34	117	Martin et al., 2020
sCoc spaid-like (JACSEQ010000039.1)	48.7	80	137	216	21	100	5.71E-21	73.9	Vera-Ponce León et al., 2021
sWSS spaid-like (2132.146.peg.209)	43.0	179	45	223	18	186	2.08E-34	116	Yeoman et al., 2019
sWSS spaid-like (2132.146.peg.1)	40.9	105	126	229	56	158	4.11E-16	62.8	
sWSS spaid-like (2132.146.peg.21)	46.0	76	159	234	1	74	5.59E-16	59.3	
sWSS spaid-like (2132.146.peg.255)	33.3	120	107	220	17	126	2.93E-12	53.5	
sWSS spaid-like (2132.146.peg.305)	45.4	99	96	194	176	267	4.81E-20	78.6	
sWSS spaid-like (2132.146.peg.596)	34.3	233	69	266	16	248	2.33E-29	105	
sWSS spaid-like (2132.146.peg.469)	33.7	237	66	300	12	219	7.15E-25	96.7	

**TABLE 4:** Homology between ankyrin genes of two *Spiroplasma ixodetis* strains based on BLASTn search.

*Spiroplasma ixodetis* gene (length)	Strain sHm ankyrin gene (length)	Identity	Aligned length	*Spiroplasma ixodetis* genes		*s*Hm genes		*e*-value	Bit score	References
								
				Start (nt)	End (nt)	Start (nt)	End (nt)			
sCoc (JACSEQ010000039.1) (303 nt)	SHM_18920 (1,971 nt)	97	300	4	303	594	295	3.92E-144	505	Vera-Ponce León et al., 2021
sWSS (2132.146.peg.596) (894 nt)	SHM_18920 (1,971 nt)	97.6	894	1	894	1	894	0	1535	Yeoman et al., 2019
sWSS (2132.146.peg.469) (1656 nt)	SHM_21210 (1,370 nt)	96.2	974	114	1,087	414	1,380	0	1,587	
sWSS (2132.146.peg.209) (558 nt)	SHM_12270 (459 nt)	85.7	385	1	383	1	383	2.78E-113	403	
sWSS (2132.146.peg.21) (276 nt)	SHM_12270 (459 nt)	96.8	158	1	158	265	422	6.55E-72	265	
sWSS (2132.146.peg.255) (723 nt)	SHM_14030 (249 nt)	97.2	221	1	221	1	221	7.94E-105	375	
sWSS (2132.146.peg.305) (966 nt)	SHM_05900 (279 nt)	90.4	220	671	890	77	279	4.01E-74	274	
sDa (SDA_03750) (162 nt)	SHM_00770 (177 nt)	97.5	162	1	162	16	177	1.33E-75	278	Martin et al., 2020
sDa (SDA_06930) (438 nt)	SHM_05900 (279 nt)	87.9	241	269	506	56	279	4.73E-72	267	
sDa (SDA_19590) (348 nt)	SHM_08510 (1,113 nt)	94.6	546	1	546	1	544	0	846	
sDa (SDA_19580) (249 nt) *NANK	SHM_08510 (1,113 nt)	95.9	249	249	1	637	884	1.46E-112	403	
sDa (SDA_10430) (978 nt)	SHM_12110 (978 nt)	98.1	978	1	978	1	978	0	1,707	
sDa (SDA_08770) (723 nt)	SHM_12270 (459 nt)	94.1	292	1	292	1	290	3.45E-125	444	
sDa (SDA_12020) (723 nt)	SHM_14030 (249 nt)	96.8	221	1	221	1	221	3.10E-103	370	
sDa (SDA_05330) (591 nt)	SHM_18920 (1,971 nt)	87.7	236	50	285	1,082	1,314	7.66E-73	272	
sDa (SDA_05340) (538 nt)	SHM_21210 (1,370 nt)	94.9	736	1	736	1	730	0	1,149	
sDa (SDA_01840) (876 nt)	SHM_28080 (693 nt)	89.8	690	49	735	1	690	0	883	
sDa (SDA_01841) (231 nt)	SHM_28070 (162 nt)	93.8	162	1	162	1	161	4.40E-65	243	
sDis (WP_252318998.1_58) (738 nt)	SHM_12270 (459 nt)	85.974	385	1	383	1	383	7.99E-115	409	Pollmann et al., 2022
sDis (WP_252319959.1_15) (648 nt)	SHM_18920 (1,971 nt)	97.651	596	1	596	1	596	0	1,024	
sDis (WP_252320693.1_6) (975 nt)	SHM_08510 (1,113 nt)	87.514	913	1	904	1	910	0	1,044	
sDis (WP_252321112.1_1) (483 nt)	SHM_21210 (1,370 nt)	96.312	461	1	461	1	461	0	758	
sDis (WP_252319264.1_36, Epsilon-like toxin) (948 nt) *NANK	SHM_25300 (495 nt) *NAK	99.187	492	154	645	1	492	0.00E + 00	887	

*NANK, non-ankyrin genes.

**FIGURE 3:**
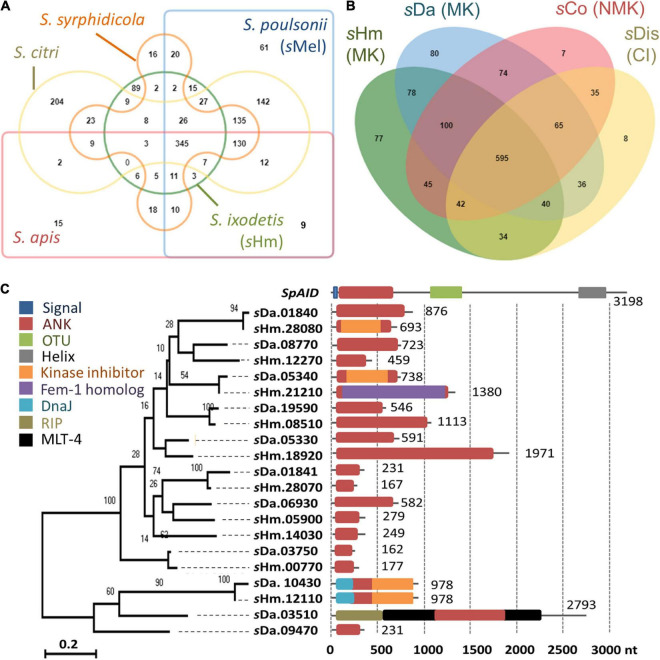
Comparison of *Spiroplasma* genomes and ankyrin genes. **(A–B)** Protein clusters conserved within *Spiroplasma* species (*S. apis, S. citri, S. ixodetis, S. poulsonii*, *and S. syrphidicola*) **(A)** and within *S*. *ixodetis* strains (*s*Hm, *s*Da, *s*Coc, and *s*Dis) **(B)**. Venn diagrams were visualized using OrthoVenn2. The *s*Hm-specific protein clusters and their functions are shown in Supplementary Table 1. MK, male-killing strain; NMK, non-male-killing strain; CI, cytoplasmic incompatibility strain. **(C)** Phylogeny of ankyrin genes in *Spiroplasma* strains *s*Hm and *s*Da. Numbers indicates nucleotide length (nt). Other domains of the genes are highlighted in different colors. Red: ankyrin; Blue: signal peptide; Green: OTU; Gray: transmembrane helix; Orange: cyclin-dependent kinase inhibitor; Purple: ubiquitination associated FEM1A_DROME; Sky blue: DNA J; Khaki: Ribosome–inactivating protein (RIP); Black: molting protein (MLT-4).

We then focused on genes conserved among *Spiroplasma* strains. Distantly related *Spiroplasma* species such as *S. ixodetis* (*s*Hm), *S. poulsonii* (*s*Mel), *S. apis* B31, *S. citri* BLH-MB, and *S. syrphidicola* EA-1 shared 345 protein clusters (Figure 3A). For *S. ixodetis* strains, two MK strains (*s*Hm and *s*Da) and two non-MK strains (*s*Coc and *s*Dis) shared 595 protein clusters (Figure 3A). In addition, MK strains *s*Hm and *s*Da possessed additional 219 conserved protein clusters. *s*Hm also harbored strain-specific 77 protein clusters (470 genes) associated with metabolism and transposition (Figure 3B and Supplementary Table 1) as well as many putative Type IV secretory system effector genes (*n* = 144, based on T4SEpre prediction at EffectiveDB, Supplementary Table 1), some of which were located in the prophage regions. In *Spiroplasma*, RIP toxin irreversibly inactivates eukaryotic cytosolic ribosomes (Hamilton et al., 2016; Ballinger et al., 2019; Garcia-Arraez et al., 2019). Based on our blast searches, RIP-4 encoded by *Spiroplasma* endosymbiont of *Drosophila neotestacea* (ASM46790.1) showed low homology to SHM_22560 (79–286 aa, *e*-value 6.7E-13, bit-score 60.8). Besides, SHM_22560 (hypothetical protein, 788 aa, Supplementary Table 1) was predicted to contain a RIP domain based on Interpro (hit: IPR016138, aligned length: 111–286 aa) and HHpred searches [hit: Sapolin (ID: 3HIQ), aligned length: 105–344 aa, *e*-value: 2.3E-29]. A homolog of an epsilon-like toxin (WP_252319264.1_36) encoded by CI-inducing *s*Dis was detected in the *s*Hm genome (SHM_25300, Table 4), while AbiEii abortive infection toxin (WP_252320055.1_19) and OTU-like cysteine protease (WP_252320277.1_1) were not detected.

Although *spaid* gene is the only ankyrin-coding gene identified in the genome of strain *s*Mel (Harumoto and Lemaitre, 2018), *s*Hm carried 10 ankyrin genes (Figure 3C). Some ankyrin genes harbored additional domains, such as those encoding for DnaJ (SHM_12110), cyclin-dependent kinase inhibitor (SHM_12110 and SHM_28080), and the protein ubiquitination associated FEM1A_DROME (SHM_21210); however, they did not encode for signal peptides, ovarian tumor-like deubiquitinase (OTU), or helix domains found in the *spaid* gene of strain *s*Me1 (Paredes et al., 2015; Harumoto and Lemaitre, 2018; Masson et al., 2018; Gerth et al., 2021). *Wolbachia* induces CI by the CI-inducing factors (Cif) harboring ankyrin repeats in insects (LePage et al., 2017). Pollmann et al. (2022) reported that CI-inducing *s*Dis strain did not harbor the *cif* gene. Similarly, *s*Hm-encoding 10 ankyrin genes has low homologies to those of other bacteria such as *Wolbachia* and *Rickettsia* and were not homologous to the *cif* as well as *spaid* genes. Intriguingly, MK *s*Da and CI *s*Dis strains had 11 and 12 ankyrin genes, respectively. These findings suggest that *S. ixodetis* has similar characteristics to *Wolbachia* endosymbionts (Duplouy et al., 2013; Arai et al., 2022b) in terms of phenotypes (i.e., CI and MK) and genetic compositions (i.e., multiple ankyrin genes). Some ankyrin genes encoded by *S. poulsonii* and *Wolbachia* have been implicated in reproductive manipulation (LePage et al., 2017; Harumoto and Lemaitre, 2018), and the ankyrin genes found in the *s*Hm genome may also be involved in MK mechanisms.

### Male-killing genes of sHm are different from those of other male-killers in *Homona magnanima*

*Homona magnanima* harbors three different types of MK endosymbionts (i.e., *Spiroplasma s*Hm, Partiti-like virus OGVs, and *Wolbachia w*Hm-t strain), some of which can coinfect the same host (Arai et al., 2020; Takamatsu et al., 2021). Moreover, microbes sharing the same niche frequently exchange virulence-associated genes (Kent and Bordenstein, 2010; Wiedenbeck and Cohan, 2011). However, we found that strain *s*Hm did not harbor any gene homologous to those of MK Partiti-like virus OGVs (Fujita et al., 2021). Moreover, strain *s*Hm did not harbor *wmk* or effector genes (e.g., CifB-like) that are present on the MK-associated prophage WO*w*Hm-t76 region of strain *w*Hm-t (Arai et al., 2020, 2022b). The *wmk* gene, a candidate gene for *Wolbachia*-induced MK (Perlmutter et al., 2019, 2021; Arai et al., 2022b), possesses a helix-turn-helix (HTH) domain containing putative transcriptional regulator. Although no *wmk* homologs were identified, strain *s*Hm harbored 87 HTH domain-encoding genes, namely putative transposase (classified into IS-30, IS-3, and IS-5 type transposase, *n* = 83), a type II toxin-antitoxin system antitoxin *HipB* (SHM_ 03650), an AAA family ATPase (SHM_24830), an XRE family transcriptional regulator (SHM_17560), and a helix-turn-helix transcriptional regulator (SHM_05440). Notably, a putative transposase SHM_03660, encoded by a gene adjacent to *s*Hm-specific *HipB*-like SHM_3650, was homologous to the *Wolbachia* transcriptional regulator. Recently, Arai et al. (2022c) demonstrated that strains *s*Hm, *w*Hm-t, and OGVs affect *H. magnanima* males in different manners. Specifically, both strains *s*Hm and *w*Hm-t trigger abnormal apoptosis and interfere with sex determination in male embryos (manifested by the alteration of *doublesex* gene splicing), but only strain *w*Hm-t impairs the dosage-compensation system of the host (manifested by the alteration of the global gene expression on sex chromosomes). In contrast, the OGVs do not affect sex-determination cascades or dosage-compensation systems. These findings and our current results support the view that phylogenetically distinct microbes have independently developed different MK machinery even for the same host, i.e., *H. magnanima*. Therefore, an unknown factor in the *s*Hm genome may be responsible for the embryonic male death of *H. magnanima*.

### sHm may require high infection density to kill *Homona magnanima* males

**FIGURE 4:**
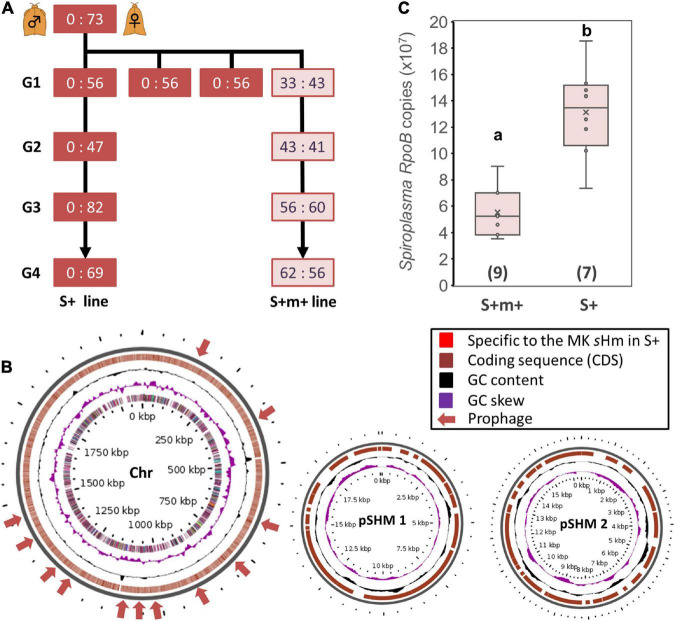
Comparison of *Spiroplasma* strain *s*Hm in MK and non-MK *Homona magnanima* lines. **(A)** *Spiroplasma*-positive 1:1 sex ratio line (S+M+ line) was maintained for over four generations (G1–G4), parallelly with the all-female S+ line. **(B)** Structural variations of the genome of *Spiroplasma* strain *s*Hm in the MK (S+) and non-MK (S+M+) *H. magnanima* lines were visualized *via* Gview software. No obvious structural variations (i.e., the red colored loci), specific to MK *s*Hm strain in the S+ matriline, were identified. Chr: main chromosome (2.1 Mb); pSHM 1: sHm plasmid 1 (20 Kb); pSHM 2: sHm plasmid 2 (16 Kb). **(C)** Abundance of *Spiroplasma* (based on copy numbers of *RpoB*) in adult females (0-day post eclosion) of S+M+ line and S+ line. The horizontal bar within the box represents the median. The upper and lower hinges of the box indicate upper quartile and lower quartile, respectively. Sample sizes (numbers of examined individuals) are indicated in parentheses. Different letters indicate significant differences between groups (Steel–Dwass test, *P* < 0.05).

We observed that one of the sublines of the MK S+ line ceased to induce MK (Figure 4A). This subline, referred to as the S+M+ line, exhibited stable *s*Hm infections for at least four generations. We simultaneously re-sequenced the genome of strain *s*Hm from S+M+ and S+ lines at the second-generation stage since their divergence. We previously demonstrated from a genomic comparison of MK *Wolbachia* (*w*Hm-t) and non-MK *Wolbachia* (*w*Hm-c) that an MK-associated 76 kb prophage region was inserted only in *w*Hm-t (Arai et al., 2022b). Similarly, we mapped the MK and non-MK *s*Hm re-sequenced Illumina reads to the complete *s*Hm genome (main chromosome and two plasmids) but did not detect any large-scale structural variation (insertions or deletions) as observed in *w*Hm-t (Figure 4B). On the other hand, we found mutations specific to the non-MK *s*Hm mutant (i.e., frameshifts or insertion of stop codons) in 21 genes encoding hypothetical proteins (*n* = 4), tyrosine-tRNA ligase (*n* = 1), and transposase (*n* = 16) (Supplementary Table 2). The 21 genes were found on the main chromosome, not in plasmids. Moreover, the *s*Hm density in the S+M+ line was lower than that in the S+ line (Steel–Dwass test, *P* < 0.05, Figure 4C).

Rapid genetic evolution leading to resistance against MK *Spiroplasma* has been reported in various hosts, such as the lacewing (Hayashi et al., 2018) and the planthopper (Yoshida et al., 2021). However, we can exclude the host genetic changes from the possible cause of the observed loss of MK phenotype because females of the S+ and S+M+ lines were parallelly mated with the males of the genetically homogeneous NSR line that had been maintained *via* inbreeding in the laboratory for over 10 years (>120 generations).

*Spiroplasma*-induced phenotypic changes have been repeatedly observed in previous studies. For example, spontaneous loss of MK was found in *S. poulsonii* strains of *Drosophila* flies, wherein substitutions and deletions occurred in the MK gene *spaid* (Harumoto and Lemaitre, 2018). Moreover, the MK strain *S. poulsonii* NSRO and its non-MK variant NSRO-A exhibit difference in bacterial densities in *D. melanogaster* (Anbutsu and Fukatsu, 2003). Indeed, bacterial density is one of the crucial factors for *Spiroplasma*- and *Wolbachia*-induced MK phenotype (Hurst and Jiggins, 2000; Kageyama et al., 2007; Arai et al., 2020). Based on these results, we speculate that the loss of MK phenotype of *s*Hm-infected *H. magnanima* was due to (i) reduced *s*Hm density and/or (ii) mutations in *s*Hm MK gene(s) or factors regulating MK gene expression levels. However, we still do not know how the small genomic rearrangements (i.e., inversions and insertions) detected in this study are involved in the phenotypic changes of *s*Hm. Future *de novo* genome construction of *s*Hm from S+M+ lines and gene function analysis would help in elucidating MK mechanisms.

### Population dynamics and tissue tropism of sHm

**FIGURE 5:**
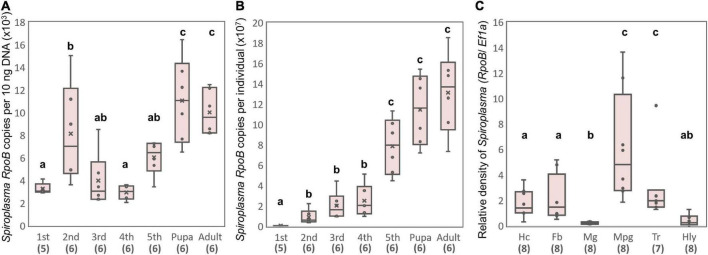
Propagation and localization of strain *s*Hm in *H. magnanima*. *Spiroplasma* densities deduced from *RpoB* copy numbers per 10 ng DNA **(A)** and *Spiroplasma* densities deduced from *RpoB* copy numbers per individual **(B)** at each developmental stage. **(C)** Relative *Spiroplasma* density in each organ deduced from *Spiroplasma RpoB* copy numbers per host *Ef1*α copy. Hc, hemocyte; Fb, fat body; Mg, midgut; Mpg, Malpighian tubules; Tr, trachea; Hly, hemolymph. Different letters indicate significant differences between groups (Steel–Dwass test, *P* < 0.05).

Strain *s*Hm was abundant at the late-developmental stages of *H. magnanima* (Figure 5A), and *s*Hm densities drastically increased from pupal to adult stages of the insect (Figure 5B). In *D. melanogaster*, *S. poulsonii* copy numbers gradually increase as the host larval development proceeds and are generally higher in pupae than in larvae (Anbutsu and Fukatsu, 2003). In contrast to *S. poulsonii*, which is reported to be the most abundant in hemolymph (Anbutsu and Fukatsu, 2003, 2006), strain *s*Hm exhibited low density in the hemolymph and high density in Malpighian tubules in the fifth instar larva stage (Figure 5C). High titers in Malpighian tubules are also a characteristic of *Wolbachia*; *Wolbachia* present in Malpighian tubules protects the host from RNA-virus infections and may constitute a secondary pool of vertically infected bacteria (Faria and Sucena, 2013; Pietri et al., 2016). The localization of strain *s*Hm in somatic tissues may have contributed to the fitness of *H. magnanima* Although there have been no reports of *S. ixodetis* localization patterns in insects, our findings suggest that *S. poulsonii* and *S. ixodetis* have distinct proliferation strategies. The hemolymph is a nutrient-rich environment but is likely an extreme habitat for microorganisms because it is well-defended by the immune system of the host (Blow and Douglas, 2019). Indeed, only a few microbial taxa are known to persist in the hemolymph of insects for extended periods without causing insect morbidity and death (Blow and Douglas, 2019). Intriguingly, *S. poulsonii s*Mel encoded more metabolic genes in its genome than *S. ixodetis s*Hm (Supplementary Table 3). Hemolymph-inhabiting *S. poulsonii* may have developed specific adaptations for its habitat, which are distinct from those of *S. ixodetis*. Further characterization of genomic features and localization patterns of *Spiroplasma* strains will clarify the distinct proliferation strategies of the two species (e.g., nutrient requirements).

### Proliferation of sHm in insect cell culture

**FIGURE 6:**
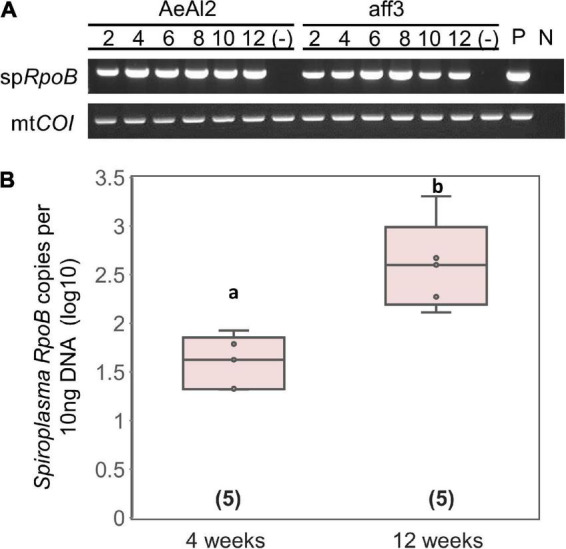
Transinfection of *Spiroplasma* strain *s*Hm into mosquito and silkworm cell lines. **(A)** Detections of strain *s*Hm in passaged cells. Numbers indicate periods (weeks) from the transinfection. P (positive control): S + female; N (negative control): NSR female. **(B)** *Spiroplasma* density in AeAl2 cells deduced from *RpoB* copy numbers per 10 ng DNA. Different letters indicate significant differences between groups (Steel–Dwass test, *P* < 0.05).

Tsugeno et al. (2017) reported that *s*Hm is horizontally transmitted by inoculating non-infected *H. magnanima* with concentrated hemolymph collected from *s*Hm-infected *H. magnanima*. Moreover, we revealed that *S. ixodetis s*Hm exhibited *Wolbachia*-like genetic characteristics (i.e., multiple ankyrin genes) and localization patterns in somatic tissues. *Wolbachia* can infect and be maintained stably in insect cell lines derived from insect taxa that are distantly related to their native hosts (Fallon, 2021). To examine whether *s*Hm can infect insect cells, we transinfected *s*Hm to the cell lines of *A. albopictus* (AeAl2) and *B. mori* (aff3), which are known to be susceptible to *Wolbachia*. *s*Hm proliferated successfully by placing a piece of fat bodies or Malpighian tubules derived from an S+ female larva into a flask containing the AeAl2 or aff3 cells (Figure 6). *s*Hm was stably maintained in the cell lines for 12 weeks (Figure 6A) but not in cell-free medium IPL-41. qPCR revealed that *s*Hm titers in AeAl2 cells were significantly higher at 12 weeks than at 4 weeks after the introduction of *s*Hm (Figure 6B). This implies the potential of *s*Hm to survive in a wide host range besides *Homona* (Tsugeno et al., 2017), such as other lepidopteran and dipteran insects. *S. ixodetis* strains isolated from Japanese ticks were also shown to be culturable in the *A. albopictus* cell line C6/36 (Thu et al., 2019). We hypothesize that *S. ixodetis* strains have a broad host range like that of *Wolbachia*. It is not clear whether *S. poulsonii* has a broad host range because there is no but one report by Hackett et al. (1986) that showed the infectivity of strain WSRO (derived from *D. willistoni*) in the *Trichoplusia ni* cell line IPLB-TN-R^2^. Several attempts to transinfect *S. poulsonii* (strain NSRO; derived from *D. nebulosa*) and MK *Spiroplasma* (derived from the lacewing *Mallada desjardinsi*) into AeAl2 and aff3 cells failed (personal observation by DK). It has been shown by hemolymph injection that *S. poulsonii* can infect drosophilid flies but not houseflies, suggesting its narrow host range (Williamson and Poulson, 1979).

### Summary and perspectives

In this study, we sequenced and analyzed the genome of an MK *S. ixodetis* strain *s*Hm. *S. poulsonii* possesses the Spaid toxin as the MK factor, whereas our study revealed that MK *S. ixodetis* did not harbor *spaid* homologs. We speculate that MK *S. ixodetis* strains found in a diverse range of insects (Hurst et al., 1999; Jiggins et al., 2000; Simon et al., 2011; Tabata et al., 2011; Sanada-Morimura et al., 2013) harbor yet-unknown MK gene(s), other than *spaid*; thus, future studies should focus on the identification of these MK genes. Besides, high infection efficiencies of strain *s*Hm in other insect cells led us to speculate that MK *S. ixodetis* has been horizontally transmitted among insect species, like *Wolbachia*, which has expanded its host range (Zhou et al., 1998; Baldo et al., 2006). Further studies would be required to understand whether closely related MK *Spiroplasma* strains (i.e., the *S. ixodetis* group) share common or different MK mechanisms, which will answer evolutionary questions such as how frequent novel MK genes arose, how MK genes moved between different *Spiroplasma* strains (if it did), and whether MK genes are associated with host sex determining systems.

## Data availability statement

All sequence data are available at DRA under BioProject: PRJDB14468, Biosamples: SAMD00547685, SAMD00547900, and DRA014961. Spiroplasma genome data are available in the DDBJ database under the following accession numbers: AP026933–AP026935.

## Author contributions

HA conducted all experiments, data analysis, and wrote the original manuscripts. MI assisted insect rearing, experiments, and discussions. DK managed the experiments and revised the original manuscript. All authors approved the final version of the manuscript.

## Footnotes

1
https://www.ebi.ac.uk/interpro/

2
https://orthovenn2.bioinfotoolkits.net/home

3
https://server.gview.ca

4
https://www.bv-brc.org

5
https://www.r-project.org/

## Abbreviations

CI: cytoplasmic incompatibility
FBS: fetal bovine serum
HTH: helix-turn-helix
JSPS: Japan Society for the Promotion of Science
MK: male-killing
OGV: Osugoroshivirus
OTU: operational taxonomic unit
RIP: ribosome-inactivating protein
WGA: whole genome amplification.
